# Protein hydrolysates in animal nutrition: Industrial production, bioactive peptides, and functional significance

**DOI:** 10.1186/s40104-017-0153-9

**Published:** 2017-03-07

**Authors:** Yongqing Hou, Zhenlong Wu, Zhaolai Dai, Genhu Wang, Guoyao Wu

**Affiliations:** 1 Hubei Key Laboratory of Animal Nutrition and Feed Science, Hubei Collaborative Innovation Center for Animal Nutrition and Feed Safety, Wuhan Polytechnic University, Wuhan, 430023 China; 2 College of Animal Science and Technology, China Agricultural University, Beijing, China; 3 Research and Development Division, Shanghai Gentech Industries Group, Shanghai, China 201015; 4 Department of Animal Science, Texas A&M University, College Station, TX USA 77843

**Keywords:** Animals, Nutrition, Peptides, Protein hydrolysates, Sustainability

## Abstract

Recent years have witnessed growing interest in the role of peptides in animal nutrition. Chemical, enzymatic, or microbial hydrolysis of proteins in animal by-products or plant-source feedstuffs before feeding is an attractive means of generating high-quality small or large peptides that have both nutritional and physiological or regulatory functions in livestock, poultry and fish. These peptides may also be formed from ingested proteins in the gastrointestinal tract, but the types of resultant peptides can vary greatly with the physiological conditions of the animals and the composition of the diets. In the small intestine, large peptides are hydrolyzed to small peptides, which are absorbed into enterocytes faster than free amino acids (AAs) to provide a more balanced pattern of AAs in the blood circulation. Some peptides of plant or animal sources also have antimicrobial, antioxidant, antihypertensive, and immunomodulatory activities. Those peptides which confer biological functions beyond their nutritional value are called bioactive peptides. They are usually 2–20 AA residues in length but may consist of >20 AA residues. Inclusion of some (e.g. 2–8%) animal-protein hydrolysates (e.g., porcine intestine, porcine mucosa, salmon viscera, or poultry tissue hydrolysates) or soybean protein hydrolysates in practical corn- and soybean meal-based diets can ensure desirable rates of growth performance and feed efficiency in weanling pigs, young calves, post-hatching poultry, and fish. Thus, protein hydrolysates hold promise in optimizing the nutrition of domestic and companion animals, as well as their health (particularly gut health) and well-being.

## Background

A protein is a macromolecule usually consisting of twenty different amino acids (AAs) linked via peptide bonds. Selenoproteins contain selenocysteine as a rare AA, but no free selenocysteine is present in animal cells. Protein is a major component of animal tissues (e.g., skeletal muscle, mammary glands, liver, and the small intestine) and products (e.g., meat, milk, egg, and wool). For example, the protein content in the skeletal muscle of growing beef cattle or pigs is approximately 70% on a dry-matter basis [1]. Thus, adequate intake of dietary protein is essential for maximum growth, production performance, and feed efficiency in livestock, poultry and fish. After being consumed in a meal by animals, the proteins in feed ingredients (e.g., blood meal, meat & bone meal, intestine-mucosa powder, fish meal, soybean meal, peanut meal, and cottonseed meal) are hydrolyzed into small peptides (di- and tri-peptides) and free AAs by proteases and oligopeptidases in the small intestine [2]; however, the types of resultant peptides can vary greatly with the physiological conditions of the animals and the composition of their diets. To consistently manufacture peptides from the proteins of animal and plant sources, robust chemical, enzymatic or microbial methods have been used before feeding to improve their nutritional quality and reduce any associated anti-nutritional factors [3, 4]. The last two methods can also improve the solubility, viscosity, emulsification, and gelation of peptides.

In animal production, high-quality protein is not hydrolyzed as feed additives. Only animal byproducts, brewer’s byproducts, and plant ingredients containing anti-nutritional factors are hydrolyzed to produce peptides for animal feeds. Proteases isolated from various sources (including bacteria, plants, and yeast) are used for the enzymatic method, whereas intact microorganisms are employed for culture in the microbial approach. To date, protein hydrolysates have been applied to such diverse fields as medicine, nutrition (including animal nutrition), and biotechnology [5]. The major objectives of this article are to highlight enzyme- and fermentation-based techniques for the industrial preparation of protein hydrolysates and to discuss the nutritional and functional significance of their bioactive peptides in animal feeding.

### Definitions of amino acids, peptides, and protein

Amino acids are organic substances that contain both amino and acid groups. All proteinogenic AAs have an α-amino group and, except for glycine, occur as L-isomers in animals and feedstuffs. A peptide is defined as an organic molecule consisting of two or more AA residues linked by peptide bonds [2]. The formation of one peptide bond results in the removal of one water molecule. In most peptides, the typical peptide bonds are formed from the α-amino and α-carboxyl groups of adjacent AAs. Peptides can be classified according to the number of AA residues. An oligopeptide is comprised of 2 to 20 AA residues. Those oligopeptides containing ≤ 10 AA residues are called small oligopeptides (or small peptides), whereas those oligopeptides containing 10 to 20 AA residues are called large oligopeptides (or large peptides). A peptide, which contains ≥ 21 AA residues and does not have a 3-dimensional structure, is termed a polypeptide [6]. A protein consists of one or more high-molecular-weight polypeptides.

**Fig. 1:**
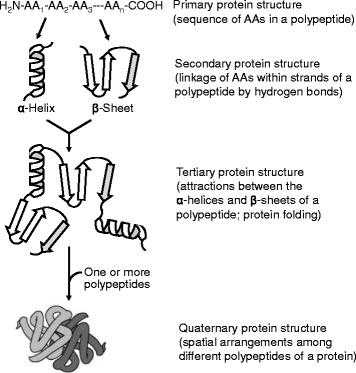
The four orders of protein structures. A protein has (1): a primary structure (the sequence of AAs along the polypeptide chain; (2) a secondary structure (the conformation of the polypeptide backbone); (3) a tertiary structure (the three-dimensional arrangement of protein); and (4) a quaternary structure (the spatial arrangement of polypeptide subunits). The primary sequence of AAs in a protein determines its secondary, tertiary, and quaternary structures, as well as its biological functions

The dividing line between proteins and polypeptides is usually their molecular weight. Generally speaking, polypeptides with a molecular weight of ≥ 8,000 Daltons (i.e., ≥ 72 AA residues) are referred to as proteins [6]. For example, ubiquitin (a single chain of 72 AA residues) and casein α-S1 (200 AA residues) are proteins, but glucagon (29 AA residues) and oxytocin (9 AA residues) are peptides. However, the division between proteins and peptides simply on the basis of their molecular weights is not absolute. For example, insulin [51 AA residues (20 in chain A and 31 in chain B)] is well recognized as a protein because it has the defined 3-dimensional structure exhibited by proteins. In contrast, PEC-60 (a single chain of 60 AA residues) [7] and dopuin (a single chain of 62 AA residues) [8], which are isolated from the pig small-intestinal mucosae, are called polypeptides. Figure 1 illustrates the four orders of protein structures (1): primary structure (the sequence of AAs along the polypeptide chain; (2) secondary structure (the conformation of the polypeptide backbone); (3) tertiary structure (the three-dimentional arrangement of protein); and (4) quaternary structure (the spatial arrangement of polypeptide subunits). The primary sequence of AAs in a protein determines its secondary, tertiary, and quaternary structures, as well as its biological functions. The forces stabilizing polypeptide aggregates are hydrogen and electrostatic bonds between AA residues.

Trichloroacetic acid (TCA; the final concentration of 5%) or perchloric acid (PCA; the final concentration of 0.2 mol/L) can fully precipitate proteins, but not peptides, from animal tissues, cells, plasma, and other physiological fluids (e.g., rumen, allantoic, amniotic, intestinal-lumen fluids, and digesta) [9, 10]. Ethanol (the final concentration of 80%) can effectively precipitate both proteins and nucleic acids from aqueous solutions [11]. This method may be useful to remove water-soluble inorganic compounds (e.g., aluminum) from protein hydrolysates. Of note, 1% tungstic acid can precipitate both proteins and peptides with ≥ 4 AA residues [10]. Thus, PCA or TCA can be used along with tungstic acid to distinguish small and large peptides.

### Industrial production of protein hydrolysates

#### General considerations of protein hydrolysis

**Fig. 2:**
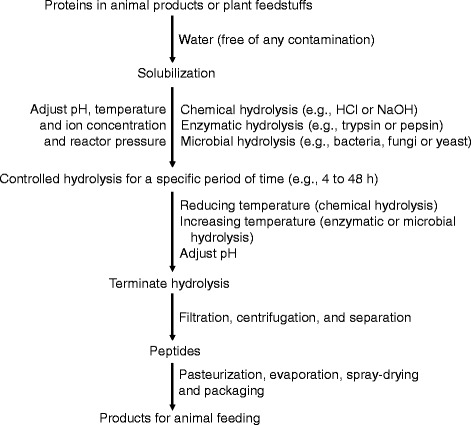
General procedures for the production of peptides from animal and plant proteins. Peptides (including bioactive peptides) can be produced from proteins present in animal products (including by-products) or plant-source feedstuffs material (e.g., soybeans and wheat) through chemical, enzymatic, or microbial hydrolysis. These general procedures may need to be modified for peptide production, depending on protein sources and product specifications

The method of choice for the hydrolysis of proteins depends on their sources. For example, proteins from feathers, bristles, horns, beaks or wool contain the keratin structure and, therefore, are usually hydrolyzed by acidic or alkaline treatment, or by bacterial keratinases [3]. In contrast, animal products (e.g., casein, whey, intestine, and meat) and plant ingredients (e.g., soy, wheat, rice, pea, and cottonseed proteins) are often subject to general enzymatic or microbial hydrolysis [4, 5]. The hydrolysis of proteins by cell-free proteases, microorganisms, acids, or bases results in the production of protein hydrolysates. The general procedures are outlined in Fig. 2. Depending on the method used, the hydrolysis times may range from 4 to 48 h. In cases where bacteriostatic or bactericidal preservatives (e.g., benzoic acid) are used in the prolonged hydrolysis of proteins by enzymes or microorganisms, the hydrolysis is usually terminated by heating to deactivate the enzyme or enzyme systems. After hydrolysis, the insoluble fractions are separated from the protein hydrolysates with the use of a centrifuge, a filter (e.g., with a 10,000 Dalton molecular weight cut-off), or a micro filtration system [3]. The filtration process is often repeated several times to obtain a desirable color and clarity of the solution. Charcoal powder is commonly used to decolorize and remove haze-forming components. If very low concentrations of salts are desired, the filtrate may be subjected to exchange chromatography to remove the salts. After filtration, the protein hydrolysate product is heat-treated (pasteurized) to kill or reduce the microorganisms. Finally, the product is dried and packaged.

#### Degree of hydrolysis

The protein hydrolysates include free AAs, small peptides, and large peptides. The proportions of these products vary with the sources of proteins, the quality of water, the type of proteases, and the species of microbes. The degree of hydrolysis, i.e., the extent to which the protein is hydrolyzed, is measured by the number of peptide bonds cleaved, divided by the total number of peptide bonds in a protein and multiplied by 100 [3]. The number of peptide bonds cleaved is measured by the moles of free AAs plus the moles of TCA- or PCA-soluble peptides. Due to the lack of standards for all the peptides generated from protein hydrolysis, it is technically challenging to quantify peptides released from animal-, plant- or microbial-sources of proteins. The percentage of AAs in the free form or the peptide form is calculated as follows:

$$ \mathrm{Percentage}\kern0.5em \mathrm{of}\kern0.5em \mathrm{A}\mathrm{A}\mathrm{s}\kern0.5em \operatorname{in}\kern0.5em \mathrm{the}\kern0.5em \mathrm{free}\kern0.5em \mathrm{form}\kern0.5em \left(\%\right)=\left(\mathrm{Total}\;\mathrm{free}\kern0.5em \mathrm{A}\mathrm{A}\mathrm{s}/\mathrm{Total}\kern0.5em \mathrm{A}\mathrm{A}\mathrm{s}\kern0.5em \mathrm{in}\kern0.5em \mathrm{protein}\right)\times 100\%; $$

$$ \mathrm{Percentage}\kern0.5em \mathrm{of}\kern0.5em \mathrm{A}\mathrm{A}\mathrm{s}\kern0.5em \mathrm{in}\kern0.5em \mathrm{peptides}\left(\%\right)=\kern0.5em \left(\mathrm{Total}\kern0.5em \mathrm{A}\mathrm{A}\mathrm{s}\kern0.5em \mathrm{in}\kern0.5em \mathrm{peptides}/\mathrm{Total}\kern0.5em \mathrm{A}\mathrm{A}\mathrm{s}\kern0.5em \mathrm{in}\kern0.5em \mathrm{protein}\right)\times 100\%; $$

When the catabolism of AAs is limited (as in enzymatic hydrolysis), the percentage of AAs in peptides is calculated as (total AAs in protein – free AAs)/total AAs in protein x 100%. High-performance liquid chromatography (HPLC) is widely used to determine free AAs [12]. HPLC and other analytical techniques (e.g., nuclear magnetic resonance spectroscopy, matrix assisted laser desorption ionization-time of flight mass spectrometry, peptide mapping, and ion-exchange chromatography) are often employed to characterize peptides in protein hydrolysates [13, 14]. When standards are available, HPLC can be used to analyze peptides.

### Methods for protein hydrolysis

#### Acid hydrolysis of proteins

Acid hydrolysis of a protein (gelatin) at a high temperature was first reported by the French chemist H. Braconnot in 1920. It is now established that the complete hydrolysis of protein in 6 mol/L HCl occurs at 110 °C for 24 h [12]. A much shorter period of time (e.g., 2 to 6 h) is used to produce peptides [3]. After the hydrolysis, the product is evaporated, pasteurized, and spray dried. The majority of acid protein hydrolysates are used as flavor enhancers (e.g., flavoring products such as hydrolyzed vegetable protein) [5]. The method of acid hydrolysis of a protein offers the advantage of low cost. However, this process results in the complete destruction of tryptophan, a partial loss of methionine, and the conversion of glutamine into glutamate and of asparagine into aspartate [5].

#### Alkaline hydrolysis of proteins

Alkaline agents, such as calcium, sodium, or potassium hydroxide (e.g., 4 mol/L), can be used at a high temperature (e.g., 105 °C) for 20 h to completely hydrolyze protein [12, 15]. Lower temperatures (e.g., 27 to 55 °C) and a shorter period of the hydrolysis time (e.g., 4 to 8 h) are often desirable for the generation of peptides in the food industry [5]. After the hydrolysis, the product is evaporated, pasteurized, and spray dried. Like acid hydrolysis of proteins, alkaline hydrolysis of proteins offers the advantage of low cost and can have a 100% recovery rate of tryptophan [12]. However, this process results in the complete destruction of most AAs (e.g., 100% loss). Thus, although alkaline hydrolysis is often used for the production of foaming agents (e.g., substitutes for egg proteins) and fire extinguisher foams, it is not widely used in the food industry.

#### Cell-free proteases

The peptide bonds of proteins can be broken down by many different kinds of proteases, which can be classified as exopeptidases and endopeptidases based on the type of reaction, namely hydrolysis of a peptide bond in the terminal region (an exopeptidase) or within an internal region (an endopeptidase) of a protein [2]. Some proteases hydrolyze dipeptides (dipeptidases), whereas others remove terminal AA residues that are substituted, cyclized, or linked by isopeptide bonds (namely peptide linkages other than those of α-carboxyl to α-amino groups; e.g., ω-peptidases). When a protease exhibits a marked preference for a peptide bond formed from a particular AA residue, the name of this AA is used to form a qualifier (e.g., “leucine” aminopeptidase and “proline” endopeptidase). In contrast, for enzymes with very complex or broad specificity, alphabetical or numerical serial names (e.g., peptidyl-dipeptidase A, peptidyl-dipeptidase B, dipeptidyl-peptidase I, and dipeptidyl-peptidase II) are employed for protein hydrolysis. Some proteases may have both exopeptidase and endopeptidase properties (e.g., cathepsins B and H). Enzymatic hydrolysis takes place under mild conditions (e.g., pH 6–8 and 30 - 60 °C) and minimizes side reactions.

Most of the cell-free enzymes for producing protein hydrolysates are obtained from animal, plant and microbial sources (Table 1). Enzymes of animal sources (particularly pigs) for protein hydrolysis are pancreatin, trypsin, pepsin, carboxylpeptidases and aminopeptidases; enzymes of plant sources are papain and bromelain; and enzymes of bacterial and fungal sources are many kinds of proteases with a broad spectrum of optimal temperatures, pH, and ion concentrations [16, 17]. The enzymes from commercial sources may be purified, semi-purified, or crude from the biological sources. The hydrolysis of proteins can be achieved by a single enzyme (e.g., trypsin) or multiple enzymes (e.g., a mixture of proteases known as Pronase, pepsin and prolidase). The choice of enzymes depends on the protein source and the degree of hydrolysis. For example, if the protein has a high content of hydrophobic AAs, the enzyme of choice would be the one that preferentially breaks downs the peptide bonds formed from these AAs. Fractionation of protein hydrolysates is often performed to isolate specific peptides or remove undesired peptides. It is noteworthy that the hydrolysis of some proteins (e.g., soy proteins and casein with papain for 18 h) can generate hydrophobic peptides and AAs with bitterness [18]. The addition of porcine kidney cortex homogenate or activated carbon to protein hydrolysates can reduce the bitterness of the peptide product [3]. Compared to acid and alkaline hydrolysis of proteins, the main advantages of enzyme hydrolysis of proteins are that: (a) the hydrolysis conditions (e.g., like temperature and pH) are mild and do not result in any loss of AAs; (b) proteases are more specific and precise to control the degree of peptide-bond hydrolysis; and (c) the small amounts of enzymes can be easily deactivated after the hydrolysis (e.g., 85 °C for 3 min) to facilitate the isolation of the protein hydrolysates. The disadvantages of enzymatic hydrolysis of protein include the relatively high cost and the potential presence of enzyme inhibitors in the raw protein materials.

**Table 1:** Proteases commonly used for protein hydrolysis

Class of enzyme	Name of enzyme	EC number	Specific cleavage
Endopeptidases			
Aspartate protease	Chymosin (rennin; pH 1.8–2)	3.4.23.4	the Phe-Met bond, clotting of milk
	Pepsin A (pH 1.8–2)	3.4.23.1	Aromatic AAs, hydrophobic AAs
Cysteine protease	Bromelain (from pieapples)	3.4.22.4	Ala, Gly, Lys, Phe, Tyr
	Cathepsin B	3.4.22.1	Arg, Lys, Phe
	Ficain (ficin; from fig tree)	3.4.22.3	Ala, Asn, Gly, Leu, Lys, Tyr, Val
	Papain (from papaya)	3.4.22.2	Arg, Lys, Phe
Metallo protease	Bacillolysin (*Bacillus* bacteria)	3.4.24.28	Aromatic AAs, Ile, Leu, Val
	Thermolysin (*Bacillus* bacteria)	3.4.24.27	Aromatic AAs, Ile, Leu, Val
Serine protease	Chymotrypsin (pH 8–9)	3.4.21.1	Aromatic AAs, Leu
	Subtilisin (from *Bacillus* bacteria)	3.4.21.14	Mainly hydrophobic AAs
	Trypsin (pH 8–9)	3.4.21.4	Arg, Lys
Exopeptidases			
Aminopeptidases	Aminopeptidase^a^	3.4.11.1	AA at the N-terminus of protein/peptide
	Aminopeptidase Y^b^	3.4.11.15	Lys at the N-terminus of protein/peptide
Carboxypeptidase	Carboxypeptidase^c^	3.4.16.1	Acidic, neutral, and basic AAs
	Glycine carboxypeptidase^d^	3.4.17.4	Gly at the C-terminus of protein/peptide
	Alanine carboxypeptidase^e^	3.4.17.8	D-Ala at the C-terminus of peptide
	Carboxypeptidase S^f^	3.4.17.9	Gly at the C-terminus of protein/peptide
Dipeptidase	Dipeptidase 1^f^	3.4.13.11	A wide range of dipeptides
	Proline dipeptidase (prolidase)^a^	3.4.13.9	AA-Pro or -hydroxyproline at the C-terminus (not Pro-Pro)
	Prolyl dipeptidase^a^	3.3.13.8	Pro-AA or Hydroxyproline-AA
Endo- and exo-peptidases			
Pronase	A mixture of proteases^a^ (from *Streptomyces griseus)*	3.4.24	Acidic, neutral, and basic AAs
Other peptidases	Dipeptidyl-peptide III^f^	3.4.14.4	Release of an N-terminal dipeptide from a peptide comprising four or more AA residues, with broad specificity
	Dipeptidyl-peptidase IV^g^	3.4.14.5	Release of an N-terminal dipeptide from a peptide consisting of proline^h^

Adapted from Kunst [16] and Dixon and Webb [17]. *AA* amino acid

^a^Metallopeptidase (requiring Mn^2+^, Mg^2+^ or Zn^2+^ for activation)

^b^Metallopeptidase (requiring Co^2+^ for activation; inhibited by Zn^2+^ and Mn^2+^)

^c^Serine carboxypeptidase

^d^Strongly inhibited by Ag^+^ and Cu^2+^

^e^Metallopeptidase (requiring Mn^2+^, Mg^2+^, Zn^2+^, Ca^2+^ or Co^2+^ for activation)

^f^Metallopeptidase (requiring Zn^2+^ for activation)

^g^Serie protease

^h^AA_1_-Pro-AA_2_, where AA_2_ is neither proline nor hydroxyproline

The efficiency and specificity of protein hydrolysis differ between microbial- and animal-source proteases [19], as reported for their lipase activity [20, 21]. For example, the hydrolysis of 18 mg casein by 40 μg pancreatin (pancreatic enzymes from the porcine pancreas) for 1–2 h in a buffer solution (43 mmol/L NaCl, 7.3 mmol/L disodium tetraborate, 171 mmol/L boric acid and 1 mmol/L CaCl_2_, pH 7.4) yields the numbers and sequences of peptides differently than NS 4 proteases (from *Nocardiopsis prasina*) and NS 5 proteases (from *Bacillus subtilis*) [19]. The pancreatin exhibited the activities of trypsin (cleavage of peptide bonds from Arg and Lys sites), chymotrypsin (cleavage of peptide bonds from Phe, Trp, Tyr, and Leu), and elastase (cleavage of peptide bonds from (Ala and other aliphatic AAs). In contrast, the microbial proteases were characterized by relatively low trypsin, carboxypeptidase and elastase activities, but high chymotrypsin activity. The time course of hydrolysis is similar among the microbial and pancreatic enzymes when caseins are the substrates. However, the rates of peptide generation are higher for pancreatin than the microbial enzymes when soya protein is the substrate. The efficiency of production of peptides with a molecular weight less than 3 kDa is higher for pancreatin than the microbial enzymes when caseins are substrates, but is similar among the microbial and pancreatic enzymes when soya protein is the substrate. In contrast, the efficiency of production of peptides with a molecular weight between 3 and 10 kDa is similar among the microbial and pancreatic enzymes when caseins are substrates or when soya protein is the substrate within 1 h incubation, but is higher for the microbial enzymes than the animal-source pancreatin.

#### Microbial hydrolysis of protein

Microorganisms release proteases to hydrolyze extracellular proteins into large peptides, small peptides and free AAs. Small peptides can be taken up by the microbes to undergo intracellular hydrolysis, yielding free AAs. Microorganisms also produce enzymes other than proteases to degrade complex carbohydrates and lipids [22]. Protein fermentation is classified into a liquid- or solid-state type. Liquid-state fermentation is performed with protein substrates under high-moisture fermentation conditions, whereas the solid-state fermentation is carried out under low-moisture fermentation conditions. The low moisture level of the solid-state fermentation can help to reduce the drying time for protein hydrolysates.

Soy sauce (also called soya sauce), which originated in China in the 2^nd^ century AD, was perhaps the earliest product of protein fermentation by microorganisms [3]. The raw materials were boiled soybeans, roasted grain, brine, and *Aspergillus oryzae* or *Aspergillus sojae* (a genus of fungus). In Koji culturing, an equal amount of boiled soybeans and roasted wheat is cultured with *Aspergillus oryzae, A. sojae, and A. tamari; Saccharomyces cerevisiae (*yeasts), and bacteria, such as *Bacillus* and *Lactobacillus* species*.* Over the past two decades, various microorganisms have been used to hydrolyze plant-source proteins, such as *Lactobacillus rhamnosus* BGT10 and *Lactobacillus zeae* LMG17315 for pea proteins, *Bacillus natto* or *B. subtilis* for soybean, and fungi *A. oryzae or R. oryzae* for soybean [23–25]. Lactic acid bacteria, such as *Lactobacillus* and *Lactococcus* species, are commonly used to ferment milk products. The major advantages of fermentation are that the appropriately used microorganisms can not only break down proteins into peptides and free AAs, but can also remove hyper-allergic or anti-nutritional factors present in the matrix of the ingredients (e.g., trypsin inhibitors, glycinin, β-conglycinin, phytate, oligosaccharides raffinose and stachyose, saponins in soybeans). The disadvantages of the microbial hydrolysis of protein are relatively high costs, as well as changes in microbial activity under various conditions and, therefore, inconsistency in the production of peptides and free AAs.

### Bioactive peptides in protein hydrolysates

#### Definition

Bioactive peptides are defined as the fragments of AA sequences in a protein that confer biological functions beyond their nutritional value [25]. They have antimicrobial, antioxidant, antihypertensive, and immunomodulatory activities. These bioactive peptides are usually 2–20 AA residues in length, but some may consist of >20 AA residues [23]. Many of them exhibit common structural properties, such as a relatively small number of AAs, a high abundance of hydrophobic AA residues, and the presence of Arg, Lys, and Pro residues [24]. In animals, endogenous peptides fulfil crucial physiological or regulatory functions. For example, PEC-60 activates Na/K ATPase in the small intestine and other tissues [26]. Additionally, many intestinal peptides (secreted by Paneth cells) have an anti-microbial function [27]. Furthermore, the brain releases numerous peptides to regulate endocrine status, food intake, and behavior in animals [28].

#### Transport of small peptides in the small intestine

In the small intestine, peptide transporter 1 (PepT1) is responsible for the proton-driven transport of extracellular di- and tri-peptides through the apical membrane of the enterocyte into the cell [29]. However, due to the high activity of intracellular peptidases in the small intestine [2], it is unlikely that a nutritionally significant quantity of peptides in the lumen of the gut can enter the portal vein or the lymphatic circulation. It is possible that a limited, but physiologically significant, amount of peptides (particularly those containing an imino acid) may be absorbed intact from the luminal content to the bloodstream through M cells, exosomes, and enterocytes via transepithelial cell transport [30, 31]. Diet-derived peptides can exert their bioactive (e.g., physiological and regulatory) actions at the level of the small intestine, and the intestinally-generated signals can be transmitted to the brain, the endocrine system, and the immune system of the body to beneficially impact the whole body.

#### ACE-inhibitory peptides

The first food-derived bioactive peptide, which enhanced vitamin D-independent bone calcification in rachitic infants, was produced from casein [32]. To date, many angiotensin-I converting enzyme (ACE)-inhibitory peptides have been generated from milk or meat (Table 2). ACE removes the C-terminal dipeptide His-Leu in angiotensin I (Ang I) to form Ang II (a potent vasoconstrictory peptide), thereby conferring their anti-hypertensive effects [33]. The best examples for ACE-inhibitory peptides are Ile-Pro-Pro (IPP) and Val-Pro-Pro (VPP), both of which are derived from milk protein through the hydrolysis of neutral protease, alkaline protease or papain [34]. There is evidence that these two proline-rich peptides may partially escape gastrointestinal hydrolysis and be transported across the intestinal epithelium into the blood circulation [35]. Similarly, the hydrolysis of proteins from meat [36] and egg yolk [37] also generates potent ACE inhibitors.

**Table 2:** Antihypertensive peptides generated from the hydrolysis of animal products

Source	Protease(s)	Amino acid sequence	IC_50_, μmol/L^a^
Pig muscle myosin	Thermolysin	Ile-Thr-Thr-Asn-Pro	549
Pig muscle myosin	Pepsin	Lys-Arg-Val-Ile-Thr-Tyr	6.1
Pig muscle actin	Pepsin	Val-Lys-Arg-Gly-Phe	20.3
Pig muscle troponin	Pepsin	Lys-Arg-Gln-Lys-Tyr-Asp-Ile	26.2
Pig muscle	Pepsin + Pancreatin	Lys-Leu-Pro	500
Pig muscle	Pepsin + Pancreatin	Arg-Pro-Arg	382
Chicken muscle	Thermolysin	Leu-Ala-Pro	3.2
Chicken muscle myosin	Thermolysin	Phe-Gln-Lys-Pro-Lys-Arg	14
Chicken muscle	Thermolysin	Ile-Lys-Trp	0.21
Chicken collagen	*Aspergillus* proteases + Proteases FP, A, G and N	Gly-Ala-X-Gly-Leu-X-Gly-Pro	29.4
Cow muscle	Thermolysin + Proteinase A	Val-Leu-Ala-Gln-Tyr-Lys	32.1
Cow muscle	Thermolysin + Proteinase A	Phe-His-Gly	52.9
Cow muscle	Proteinase K	Gly-Phe-His-Ile	64.3
Cow skin gelatin	Alcalase + Pronase E + Collagenase	Gly-Pro-Val	4.67
Cow skin gelatin	Alcalase + Pronase E + Collagenase	Gly-Pro-Leu	2.55
Bonito (fish) muscle	Thermolysin	Leu-Lys-Pro-Asn-Met	2.4
Bonito (fish) muscle	Thermolysin	Leu-Lys-Pro	0.32
Bonito (fish) muscle	Thermolysin	Ile-Lys-Pro	6.9
Salmon muscle	Thermolysin	Val-Trp	2.5
Salmon muscle	Thermolysin	Met-Trp	9.9
Salmon muscle	Thermolysin	Ile-Trp	4.7
Sardine muscle	Alcalase	Ile-Tyr	10.5
Sardine muscle	Alcalase	Ala-Lys-Lys	3.13
Sardine muscle	Alcalase	Gly-Trp-Ala-Pro	3.86
Sardine muscle	Alcalase	Lys-Tyr	1.63
Alaska pollack skin	Alcalase + Pronase + Collagenase	Gly-Pro-Leu	2.65
Alaska pollack skin	Alcalase + Pronase + Collagenase	Gly-Pro-Met	17.1
Shark muscle	Protease SM98011	Glu-Tyr	1.98
Shark muscle	Protease SM98012	Phe-Glu	2.68
Shark muscle	Protease SM98013	Cys-Phe	1.45
Egg yolk	Pepsin	Tyr-Ile-Glu-Ala-Val-Asn-Lys-Val-Ser-Pro-Arg-Ala-Gly-Gln-Phe	9.4^b^
Egg yolk	Pepsin	Tyr-Ile-Asn-Gln-Met-Pro-Gln-Lys-Ser-Arg-Glu	10.1^b^

Adapted from Ryan JT et al. [28], Ryder et al. [33], and Zambrowicz et al. [34]

*“X”* hydroxyproline

^a^Inhibition of angiotensin-I converting enzyme (ACE) activity. All values are expressed as μM, except for egg yolk-derived peptides (μg/mL) as indicated by a superscript “b”

#### Antioxidative and antimicrobial peptides

**Fig. 3:**
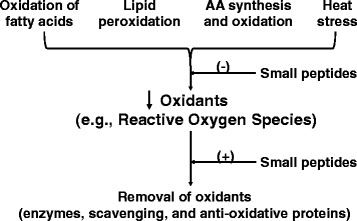
Inhibition of cellular oxidative stress by dietary small peptides in the small intestine. The small peptides, which are supplemented to the diets of animals (particularly young animals), can reduce the production of oxidants by the small intestine and enhance the removal of the oxidants, leading to a decrease in their intracellular concentrations and alleviating oxidative stress. (−), inhibition; (+), activation; ↓, decrease

Many small peptides from animal products (e.g., fish and meat) (Table 3) and plant-source feedstuffs [25] have anti-oxidative functions by scavenging free radicals and/or inhibiting the production of oxidants and pro-inflammatory cytokines [38–41]. These small peptides can reduce the production of oxidants by the small intestine, while enhancing the removal of the oxidants, resulting in a decrease in their intracellular concentrations and alleviating oxidative stress (Fig. 3). Many of the bioactive peptides have both ACE-inhibitory and anti-oxidative effects [36, 37]. Additionally, some peptides from animal (Table 4) and plant protein-hydrolysates [25] also have antimicrobial effects, as reported for certain endogenous peptides in the small intestine [27]. These antimicrobial peptides exert their actions by damaging the cell membrane of bacteria, interfering with the functions of their intracellular proteins, inducing the aggregation of cytoplasmic proteins, and affecting the metabolism of bacteria [42–44], but the underlying mechanisms remain largely unknown [27].

**Table 3:** Antioxidative peptides generated from the hydrolysis of animal proteins

Source	Protease(s)	Amino acid sequence
Pig muscle actin	Papain + Actinase E	Asp-Ser-Gly-Val-Thr
Pig muscle	Papain + Actinase E	Ile-Glu-Ala-Glu-Gly-Glu
Pig muscle tropomyosin	Papain + Actinase E	Asp-Ala-Gln-Glu-Lys-Leu-Glu
Pig muscle tropomyosin	Papain + Actinase E	Glu-Glu-Leu-Asp-Asn-Ala-Leu-Asn
Pig muscle myosin	Papain + Actinase E	Val-Pro-Ser-Ile-Asp-Asp-Gln-Glu-Glu-Leu-Met
Pig collagen	Pepsin + Papain + others^a^	Gln-Gly-Ala-Arg
Pig blood plasma	Alcalase	His-Asn-Gly-Asn
Chicken muscle	---	His-Val-Thr-Glu-Glu
Chicken muscle	---	Pro-Val-Pro-Val-Glu-Gly-Val
Deer muscle	Papain	Met-Gln-Ile-Phe-Val-Lys-Thr-Leu-Thr-Gly
Deer muscle	Papain	Asp-Leu-Ser-Asp-Gly-Glu-Gln-Gly-Val-Leu
Bovine milk casein	Pepsin, pH 2, 24 h	Tyr-Phe-Tyr-Pro-Glu-Leu
Bovine milk casein	Pepsin, pH 2, 24 h	Phe-Tyr-Pro-Glu-Leu
Bovine milk casein	Pepsin, pH 2, 24 h	Tyr-Pro-Glu-Leu
Bovine milk casein	Pepsin, pH 2, 24 h	Pro-Glu-Leu
Bovine milk casein	Pepsin, pH 2, 24 h	Glu-Leu
Bovine milk casein	Trypsin, pH 7.8, 24–28 h	Val-Lys-Glu-Ala-Met-Pro-Lys
Bovine milk casein	Trypsin, pH 7.8, 24–28 h	Ala-Val-Pro-Tyr-Pro-Gln-Arg
Bovine milk casein	Trypsin, pH 7.8, 24–28 h	Lys-Val-Leu-Pro-Val-Pro-Glu-Lys
Bovine milk casein	Trypsin, pH 7.8, 24–28 h	Val-Leu-Pro-Val-Pro-Glu-Lys
Bovine whey protein	Thermolysin, 80 °C, 8 h	Leu-Gln-Lys-Trp
Bovine whey protein	Thermolysin, 80 °C, 8 h	Leu-Asp-Thr-Asp-Tyr-Lys-Lys
Bovine β-Lactoglobulin	Corolase PP, 37 °C, 24 h	Trp-Tyr-Ser-Leu-Ala-Met-Ala-Ala-Ser-Asp-Ile
Bovine β-Lactoglobulin	Corolase PP, 37 °C, 24 h	Met-His-Ile-Arg-Leu
Bovine β-Lactoglobulin	Corolase PP, 37 °C, 24 h	Try-Val-Glu-Glu-Leu
Egg yolk	Pepsin	Tyr-Ile-Glu-Ala-Val-Asn-Lys-Val-Ser-Pro-Arg-Ala-Gly-Gln-Phe
Egg yolk	Pepsin	Tyr-Ile-Asn-Gln-Met-Pro-Gln-Lys-Ser-Arg-Glu

Adapted from Ryder et al. [33], Zambrowicz et al. [34], Shimizu and Son [35], Bah et al. [36], Memarpoor-Yazdia et al. [37], and Power et al. [38]

^a^Bovine pancreatic proteases plus bacterial proteases from *Streptomycest bacillus*

**Table 4:** Antimicrobial peptides generated from the hydrolysis of animal proteins or synthesized by intestinal mucosal cells

Source	Amino acid sequence	Gram-positive bacteria	Gram-negative bacteria
Bovine meat	Gly-Leu-Ser-Asp-Gly-Glu-Trp-Gln	*Bacillus cereus Listeria monocytogenes*	*Salmonella typhimurium Escherichia coli*
	Gly-Phe-His-Ile	No effect	*Pseudomonas aeruginosa*
	Phe-His-Gly	No effect	*Pseudomonas aeruginosa*
Bovine collagen	Peptides < 2 kDa (by collagenase)^a^	*Staphylococcus aureus*	*Escherichia coli*
Goat whey	GWH (730 Da) and SEC-F3 (1,183 Da) (hydrolysis by Alcalase)	*Bacillus cereus Staphylococcus aureus*	*Salmonella typhimurium Escherichia coli*
Red blood cells	Various peptides (24-h hydrolysis by fugal proteases)	*Staphylococcus aureus*	*Escherichia coli Pseudomonas aeruginosa*
Hen egg white lysozyme	Asn-Thr-Asp-Gly-Ser-Thr-Asp-Tyr-Gly-Ile-Leu-Gln-Ile-Asn-Ser-Arg (hydrolysis by papain and trypsin)^b^	*Leuconostoc-mesenteroides*	*Escherichia coli*
Trout by-products	Various peptides (20–30% of hydrolysis) (hydrolysis by trout pepsin)	*Renibacterium-salmoninarum*	*Flavobacterium psychrophilum*
Small intestine (Paneth cells)	α-Defensins, lysozyme C, angiogenin-4 and cryptdin-related sequence peptides	Gram-positive bacteria broad-spectrum)	Gram-negative bacteria (broad-spectrum)
	Phospholipid-*sn*-2 esterase and C-type lectin	Gram-positive bacteria (broad-spectrum)	No effect

Adapted from Lima et al. [39], Osman et al. [40], and Wald et al. [41]

^a^minimal inhibition concentrations = 0.6 – 5 mg/mL

^b^minimal inhibition concentrations = 0.36 – 0.44 μg/mL

#### Opioid peptides

The hydrolysis of certain proteins [e.g., casein, gluten (present in wheat, rye and barley), and soybeans] in the gastrointestinal tract can generate opioid peptides [45]. This can be performed in vitro by using digestive enzymes from the small intestine of mammals (e.g., pigs). Opioid peptides are oligopeptides (typically 4–8 AA residues in length) that bind to opioid receptors in the brain to affect the gut function [46, 47], as well as the behavior and food intake of animals (Table 5). Furthermore, the protein hydrolysates containing opioid-like peptides may be used as feed additives to alleviate stress, control pain and sleep, and modulate satiety in animals.

**Table 5:** Opioid peptides generated from the enzymatic hydrolysis of animal and plant proteins in the gastrointestinal tract

Source	Name of opioid peptide	Amino acid sequence
Milk casein	Bovine β-casomorphin 1–3	Tyr-Pro-Phe-OH
	Bovine β-casomorphin 1–4	Tyr-Pro-Phe-Pro-OH
	Bovine β-casomorphin 1–4, amide	Tyr-Pro-Phe-Pro-NH_2_
	Bovine β-casomorphin 5	Tyr-Pro-Phe-Pro-Gly-OH
	Bovine β-casomorphin 7	Tyr-Pro-Phe-Pro-Gly-Pro-Ile-OH
	Bovine β-casomorphin 8	Tyr-Pro-Phe-Pro-Gly-Pro-Ile-Pro-OH^a^
Gluten protein	Gluten exorphin A5	Gly-Tyr-Tyr-Pro-Thr-OH
	Gluten exorphin B4	Tyr-Gly-Gly-Trp-OH
	Gluten exorphin C	Tyr-Pro-Ile-Ser-Leu-OH
	Gliadorphin	Tyr-Pro-Gln-Pro-Gln-Pro-Phe-OH
Soybean protein	Soymorphin-5^b^	Tyr-Pro-Phe-Val-Val-OH
	Soymorphin-5, amide	Tyr-Pro-Phe-Val-Val-NH_2_
	Soymorphin-6	Tyr-Pro-Phe-Val-Val-Asn-OH
	Soymorphin-7	Tyr-Pro-Phe-Val-Val-Asn-Ala-OH
Spinach protein	Rubiscolin-5	Gly-Tyr-Tyr-Pro-OH
	Rubiscolin-6	Gly-Tyr-Tyr-Pro-Thr-OH

Adapted from Li-Chan [21], López-Barrios et al. [22], Shimizu and Son [35], Bah et al. [36], and Froetschel [42]

^a^Another form of bovine β-casomorphin 8 has histidine instead of proline in position 8, depending on whether the peptide is derived from A1 or A2 beta-casein

^b^Derived from β-conglycinin β-subunit

### Applications of plant- and animal-protein hydrolysates in animal nutrition

#### General consideration

A major goal for animal agriculture is to enhance the efficiency of feed utilization for milk, meat and egg production [48]. This approach requires optimal nutrition to support the function of the small intestine as the terminal site for the digestion and absorption of dietary nutrients [49]. To date, peptides generated from the hydrolysis of plant and animal proteins are included in the diets for feeding pigs, poultry, fish, and companion animals. The outcomes are positive and cost-effective for the improvement of intestinal health, growth and production performance [50]. The underlying mechanisms may be that: (a) the rate of absorption of small peptides is greater than that of an equivalent amount of free AAs; (b) the rate of catabolism of small peptides by the bacteria of the small intestine is lower than that of an equivalent amount of free AAs; (c) the composition of AAs entering the portal vein is more balanced with the intestinal transport of small peptides than that of individual AAs; (e) provision of functional AAs (e.g., glycine, arginine, glutamine, glutamate, proline, and taurine) to enhance anti-oxidative reactions and muscle protein synthesis [51, 52]; and (e) specific peptides can improve the morphology, motility and function of the gastrointestinal tract (e.g., secretion, motility, and anti-inflammatory reactions), endocrine status in favor of anabolism, and feed intake, compared with an equivalent amount of free AAs. In swine nutrition research, most of the studies involving the addition of peptides to diets have been conducted with post-weaning pigs to improve palatability, growth, health, and feed efficiency [53–58]. This is primarily because young animals have immature digestive and immune systems and weanling pigs suffer from reduced feed intake, gut atrophy, diarrhea, and impaired growth. Moreover, peptide products have been supplemented to the diets of calves [59], poultry [60, 61], fish [62, 63], and companion animals [64] to improve their nutrition status, gut function, and abilities to resist infectious diseases.

#### Plant peptides

As noted previously, plant-source protein ingredients often contain allergenic proteins and other anti-nutritional factors which can limit their practical use, particularly in the diets of young animals [50] and companion animals [64]. For example, soybeans can be processed to manufacture soybean meal and soybean protein concentrates for the elimination of some anti-nutritional substances. However, the soy products still contain considerable amounts of protein-type allergens (e.g., glycinin and β-conglycinin) and significant quantities of trypsin inhibitors, lectins (hemagglutinins), phytic acid, soy oligosaccharides (raffinose and stachyose), and steroid glycosides (soy saponins) [18, 24, 25]. Fermentation of soybeans by the commonly used microorganism (e.g., *Aspergillus* species, *Bacillus* species, and *Lactobacillus* species) has been reported to improve growth performance and feed efficiency in weanling pigs [50]. Thus, 3- to-7-week-old pigs fed a corn- and soybean meal-based diet containing 3% or 6% fermented soybean meal grew at a rate comparable to that of the same percentage of dried skim milk [54]. Likewise, 4.9% fermented soybean meal could replace 3.7% spray-dried plasma protein in the diets of 3- to-7-week-old pigs fed a corn- and soybean meal-based diet without affecting growth performance or feed efficiency [54]. Similar results were obtained for the Atlantic salmon fed a diet containing 40% protein from fermented soy white flakes [60]. Of interest, 50% of fish meal in the diet of juvenile red sea bream can be replaced by the same percentage of soybean protein hydrolysate [63]. The inclusion of plant-protein hydrolysate in diets is important in aquaculture because fish meal is becoming scarce worldwide. Furthermore, as a replacement of the expensive skim milk powder, the hydrolysate of soy protein isolate (19.7% in diet) can be used to sustain high growth-performance in calves [59]. Finally, acidic hydrolysates of plant proteins (e.g., wheat gluten which contains a high amount of glutamine plus glutamate), often called hydrolyzed vegetable proteins, can be included at a 1 to 2% level in the diets of companion animals to provide savory flavors due to the high abundance of glutamate in the products [64].

#### Animal peptides

Postweaning piglets fed a diet containing 6% spray-dried porcine intestine hydrolysate (SDPI; the co-product of heparin production) for 2 wk had better growth performance than those fed the control diet, the basal diet containing spray-dried plasma, or the basal diet containing dried whey [55, 56]. There was a carry-over effect on enhancing growth performance during weeks 3–5 postweaning in piglets that were previously fed the SDPI [56], which was likely due to an increased area of the intestinal villus as well as improved digestion and absorption of dietary nutrients [57]. Similarly, Stein (2002) reported that piglets (weaned at 20 days of age) fed a weanling diet containing 1.5, 3 or 4.5% SDPI had better growth performance and greater feed efficiency in comparison to piglets consuming the same amount of a fish meal-supplemented diet. Of note, these effects of the SDPI supplementation were dose-dependent. In addition, postweaning piglets fed a corn-, soybean meal-, and dried whey-based diet containing 6% enzymatically hydrolyzed proteins (from blend of swine blood and selected poultry tissues) exhibited a growth rate and a feed efficiency that were comparable to those for piglets fed a diet containing the same percentage of spray-dried blood cells [53]. Likewise, the inclusion of 2.5 5 or 7.5% hydrolyzed porcine mucosa in a corn- and soybean meal-based diet enhanced daily weight gain and nutrient retention in growing chicks [61]. Furthermore, broilers fed a diet containing 5% Atlantic salmon protein hydrolysates (from the viscera) had better growth performance than those fed a diet with or without 4% fish meal [60]. Finally, addition of the protein hydrolysate of fish by-products to the diet (at a 10% inclusion level) improved intestinal development, growth, immunological status, and survival in European sea bass larvae challenged with *Vibrio anguillarum* (a Gram-negative bacterium) [65]. Thus, SDPI or other hydrolysates of animal proteins hold promise for animal production.

### Potential scale and economic value for the global use of animal and plant protein hydrolysates in animal feeding

Industrial processing of domestic farm animals generates large amounts of tissues (30–40% of body weight) not consumed by humans, including viscera, carcass-trimmings, bone (20–30% of body weight), fat, skin, feet, small-intestinal tissue (2% of body weight), feather (up to 10% of body weight), and collectible blood (5% body weight), with the global human-inedible livestock and poultry byproducts being ~54 billion kg/yr [66–68]. Likewise, fish processing industries produce large amounts of wastes (up to 55% of body weight), such as muscle-trimmings (15–20%), skin and fins (1–3%), bones (9–15%), heads (9–12%), viscera (12–18%), and scales, with the global human-inedible fish byproducts being ~6 billion kg/yr [66–69]. Thus, the global annual volume of total animal by-products generated by the processing industries is approximately 60 billion kg annually. Assuming that only 5% of the animal by-products and plant products for feed are used for protein hydrolysis, and based on the current average prices of animal, soybean, and wheat protein hydrolysates [70], their yields are 3, 6.75 and 12.75 billion kg/yr, respectively, and their economic values are 4.5, 3.88 and 20.02 billion US $/yr (Table 6). Thus, protein hydrolysates from the by-products of pigs or poultry and from plant ingredients hold great promise in sustaining the animal agriculture and managing companion animals worldwide.

**Table 6:** Potential scale and economic values for the global use of animal and plant protein hydrolysates (PH) in animal feeding

Type	Annual global production^a^	Annual use for animal feeding^a^	Amount used for production of PH^b^	Current price^c^	Total value for PH
	Billion kg/yr	Billion kg/yr	Billion kg/yr	US $/kg	Billion US $/yr
ABP	172	60	3	1.5	4.50
Soybean	180	135	6.75	0.575	3.88
Wheat	750	255	12.75	1.57	20.02

ABP, animal byproducts (including livestock, poultry and fish)

^a^Food and Agriculture Organization [69]

^b^Assuming that 5% of the ABP or plant products for animal feeds are used to produce protein hydrolysates

^c^The prices for peptone (a representative of animal protein hydrolysates), fermented soybean, and hydrolyzed wheat protein [70].

### Future research directions

The nutritional value of protein hydrolysates as flavor enhancers, functional ingredients, and precursors for protein synthesis depends on the composition of free AAs, small peptides and large peptides in the products, as well as their batch-to-batch consistence. At present, such data are not available for the commercially available products of animal or plant hydrolysates and should be obtained with the use of HPLC and mass spectrometry. Only when the composition of protein hydrolysates is known, can we fully understand their functionally active components and the mechanisms of their actions. In addition, the net rates of the transport of small peptides across the small intestine are not known for all the protein hydrolysates currently used in animal feeding. This issue can be readily addressed with the use of Ussing chambers [71]. There is also concern that some animal protein hydrolysates, which contain a high proportion of oligopeptides with a high abundance of basic AAs, have a low palatability for animals (particularly weanling piglets), and, therefore, the inclusion of the protein hydrolysates in animal feeds may be limited. Such a potential problem may be substantially alleviated through: (a) the addition of exopeptidases and a longer period of hydrolysis to remove basic and aliphatic AAs from the C- and N-terminals of the polypeptides; and (b) appropriate supplementation with glycine, monosodium glutamate and inosine. Furthermore, the role of animal and plant protein hydrolysates in the signaling of intestinal epithelial cells and bacteria and metabolic regulation in these cells should be investigated to better understand how these beneficial products improve gut integrity, immunity, and health. Finally, the potential of protein hydrolysates as alternatives to dietary antibiotics should be explored along with studies to elucidate the underlying mechanisms. All these new lines of research will be particularly important for animals with compromised intestinal structure and function (e.g., neonates with intrauterine growth restriction and early-weaned mammals) and raised under adverse environmental conditions (e.g., high or low ambient temperatures).

## Conclusion

Plant- and animal-protein hydrolysates provide highly digestible peptides and bioactive peptides, as well as specific AAs (e.g., glutamate) to confer nutritional and physiological or regulatory functions in animals. The industrial production of these protein hydrolysates involves: (a) strong acidic or alkaline conditions, (b) mild enzymatic methods, or (c) fermentation by microorganisms. The degree of hydrolysis is assessed by the number of peptide bonds cleaved divided by the total number of peptide bonds in a protein. Chemical hydrolysis is often employed to generate savory flavors, whereas microbial fermentation not only produces peptides but also removes anti-nutritional factors in protein ingredients. In addition to their nutritional value to supply AAs, bioactive peptides (usually 2–20 AA residues in length) have antimicrobial, antioxidant, antihypertensive, and immunomodulatory roles. These peptides exert beneficial effects on improving intestinal morphology, function, and resistance to infectious diseases in animals (including pigs, calves, chickens, companion animals, and fish), thereby enhancing their health and well-being, as well as growth performance and feed efficiency. This provides a cost-effective approach to converting animal by-products, brewer’s byproducts, or plant feedstuffs into high-quality protein-hydrolysate ingredients to feed livestock, poultry, fish, and companion animals.

## Abbreviations

AA: Amino acid
ACE: Angiotensin-I converting enzyme
HPLC: High-performance liquid chromatography
PCA: Perchloric acid
PH: Protein hydrolysates
SDPI: Spray-dried porcine intestine hydrolysate
TCA: Trichloroacetic acid
